# Retrograde ureteric stent insertion from percutaneous suprapubic access to the bladder in renal transplant recipients with ureteric stenosis: a novel minimally invasive technique

**DOI:** 10.1186/s12894-020-00748-6

**Published:** 2020-11-02

**Authors:** Jian-Hui Wu, Chun-Bai Mo, Li Dong-Zhai, Fei Luo, Qing-Tong Ma, Shi-Qiang Yang

**Affiliations:** 1 Department of Urology, Tianjin First Central Hospital, No. 24 Fukang Road, Nankai District, Tianjin, 300192 China; 2 Department of Renal Transplant, Tianjin First Central Hospital, Tianjin, 300192 China; 3 Department of Anatomy and Histology, School of Basic Medical Sciences, Tianjin Medical University, Tianjin, 300070 China; 4 Department of Urology, Tianjin Union Medical Center, Tianjin, 300121 China

**Keywords:** Kidney transplantation, Ureteral obstruction, Stent, Complications, Ureteral calculi, Kidney calculi, Retrospective studies

## Abstract

**Background:**

Ureteric stricture is a common and salvaging complications after renal transplantation. Two treatment methods are usually used, retrograde ureteral stent placement and percutaneous nephrostomy. The former has a higher failure rate, the latter has a great risk. Therefore, a safe and reliable treatment is needed.

**Case presentation:**

A technique of retrograde insertion of ureteral stent was established, which was applicable in three transplant recipients with post-transplant ureteral stenosis, and the data was retrospectively recorded. The patients are 2 men and 1 woman, ages 44, 27 and 32 years. These patients underwent a total of five times of retrograde insertion of ureteral stent between 2018 and 2019. None of these patients had any postoperative complication, but all patients had complete recovery from oliguric status within two weeks.

**Conclusions:**

The retrograde ureteric stent insertion by percutaneous suprapubic access to the bladder (RUS-PSAB) was demonstrated feasibility and safety in a case series with short-term follow-up. However, larger prospective studies are needed.

## Background

Urological complications occupy 2–14% of all complications during renal transplantation [1, 2]. The incidence of ureteric stricture after kidney transplantation can reach up to 3% [2]. Among all ureteric stricture cases, 73% occur at the distal end, involving the ureteroneocystostomy [3]. When patients who received a kidney present with hydronephrosis due to obstructing stones or ureteric stricture, they should undergo the insertion of either a nephrostomy or a ureteral stent as soon as possible. Conventionally, ureteral catheterization under a cystoscope has been considered an initial approach before percutaneous renal puncture with drainage [4, 5]. Several reports have suggested that nephrostomy placement is cost-saving and safe. However, in the experience of the investigators, most candidates are reluctant to undergo percutaneous renal drainage due to hidden risks. A randomized study revealed that there was no significant difference between nephrostomy and ureteral stenting [6]. Furthermore, retrograde ureteral stent placement in transplant recipients can be time-consuming and technically difficult. In the present study, we report our experience in using retrograde ureteral stent insertion through percutaneous suprapubic access to the bladder (RUS-PSAB) to successfully relieve ureteral obstruction in three patients with confirmed allograft hydronephrosis.

## Case presentation

Patient 1 was a 44-year-old Chinese male with end-stage renal disease (ESRD) of unknown origin and a blood type of AB (Rh+). Before accepting renal transplantation from his 59-year old father with a blood type of B (Rh+), the patient has been on hemodialysis for more than one year. During surgery, the graft renal vein and artery were anastomosed to the patient’s right external iliac vein and artery, respectively, using 5-0 polypropylene in an end-to-side manner. Then, a 6-Fr 13-cm double-J stent was placed, and the transplanted ureter was anastomosed to the recipient bladder using the Lich-Gregoire technique, which incorporated a 5-0 polyglactin interrupted suture. The patient’s postoperative immunosuppression regimen consisted of tacrolimus, mycophenolate mofetil and prednisolone. Four weeks after the transplantation, the double-J stent was removed by cystoscope under local anesthesia. The patient’s serum creatinine (s-CR) at discharge was 75 μmol/L (normal range: 45–84 μmol/L).

**Fig. 1:**
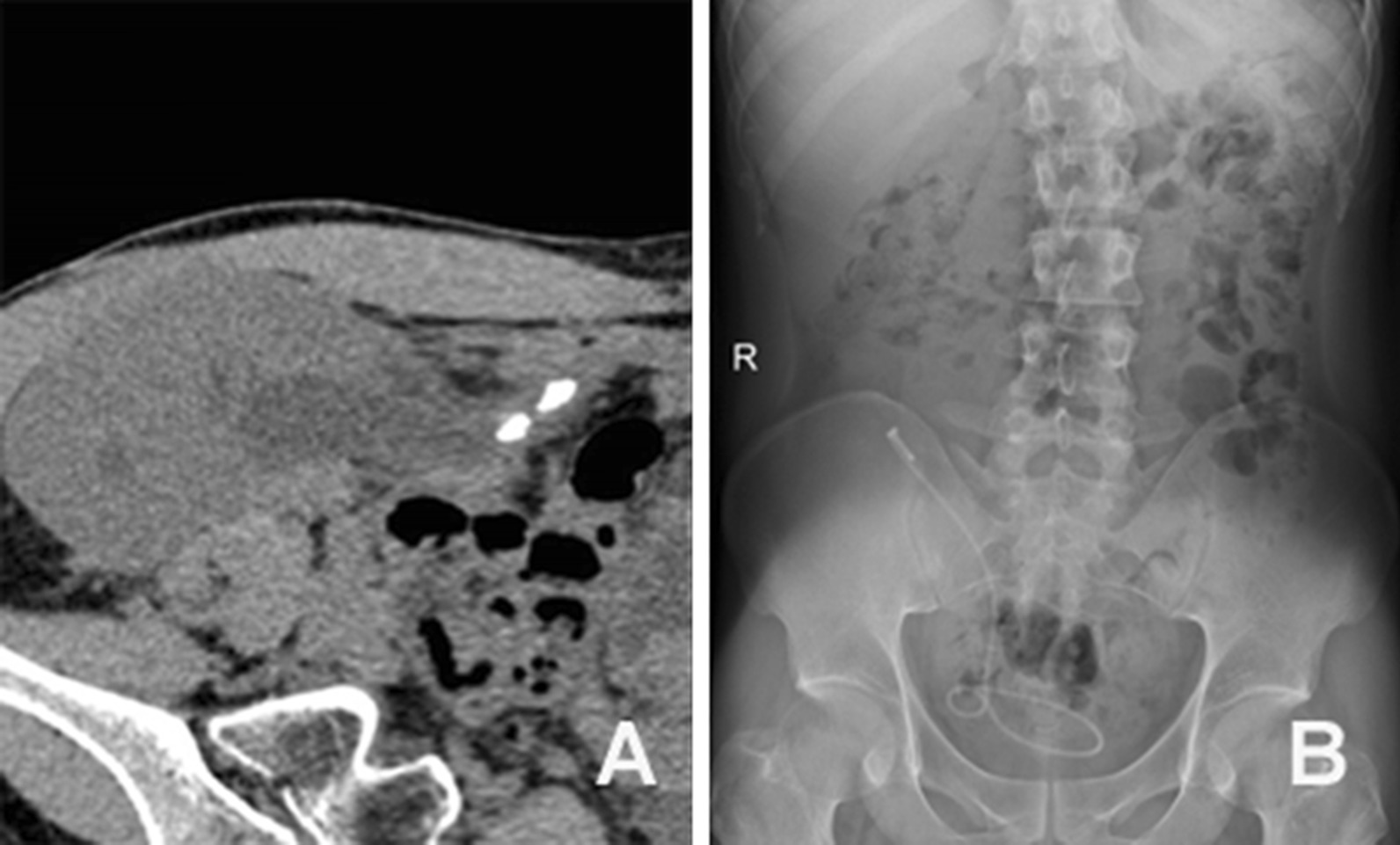
The placement of a double-J stent in a 39-year-old Chinese male developing ureterovesical junction stenosis and kidney calculi. **a** The computed tomography revealed multiple allograft stones. **b** The KUB film revealed that the double-J stent was inserted into the pelvis of the renal allograft

At five years post-transplantation, the ultrasound revealed multiple calculi at renal calices (Fig. 1). Watchful waiting was suggested, since the patient was asymptomatic with normal s-CR. After four months, the patient was admitted for swelling pain that involved the renal allograft and decreased urine output. The allograft ultrasound revealed severe hydronephrosis, and the computed tomography (CT) demonstrated a 10-mm calculus obstructing the ureterocystostomic site. The patient underwent a cystoscopy for ureteral stenting, but failed. Since the transplanted ureteral orifice was located at the dome of the bladder, which formed an oblique angle between the orifice and cystoscopic access site, the intubation from a different plane carried a low probability of success. Prior to the percutaneous nephrostomy (PCN), percutaneous cystostomy was performed at a position relative to the ureteral orifice of the renal allograft using an 18-G needle (cook), in order to correct the oblique intubation angle. Under cystoscope guidance, the channel size was expanded step by step to 10F, followed by the placement of an expanded sheath close to the edge of the transplanted ureteral orifice. One retrograde guidewire was passed into the transplanted ureteral orifice, and a double-J tube (Black Silicone Filiform DP Ureteral Stent Set Wire Guide with Hydrophilic Coating, Cook Incorporated, USA) was successfully inserted into the allograft along the guidewire. After two weeks, a retrograde ureteroscope was inserted and reached the calculus, and Holmium-yttrium aluminum garnet (Ho-YAG) laser lithotripsy was performed (VersaPulse ® PowerSuite TM 100+W, Lumenis Surgical, USA). Although the lithotripsy was successfully performed at the first time, the ureter could not be reached through the conventional route of the ureteroscope again for subsequent calculus fragment removal. After several failed attempts, the surgeons repeated the trans-vesical puncture to place the double-J tube similar to the first treatment session. After 1 month, the double-J tube was removed using a cystoscope, and there were few stone residues left in the allograft, with diameters of < 4 mm. The patient’s s-CR remained at 85 μmol/L (Fig. 1).

**Fig. 2:**
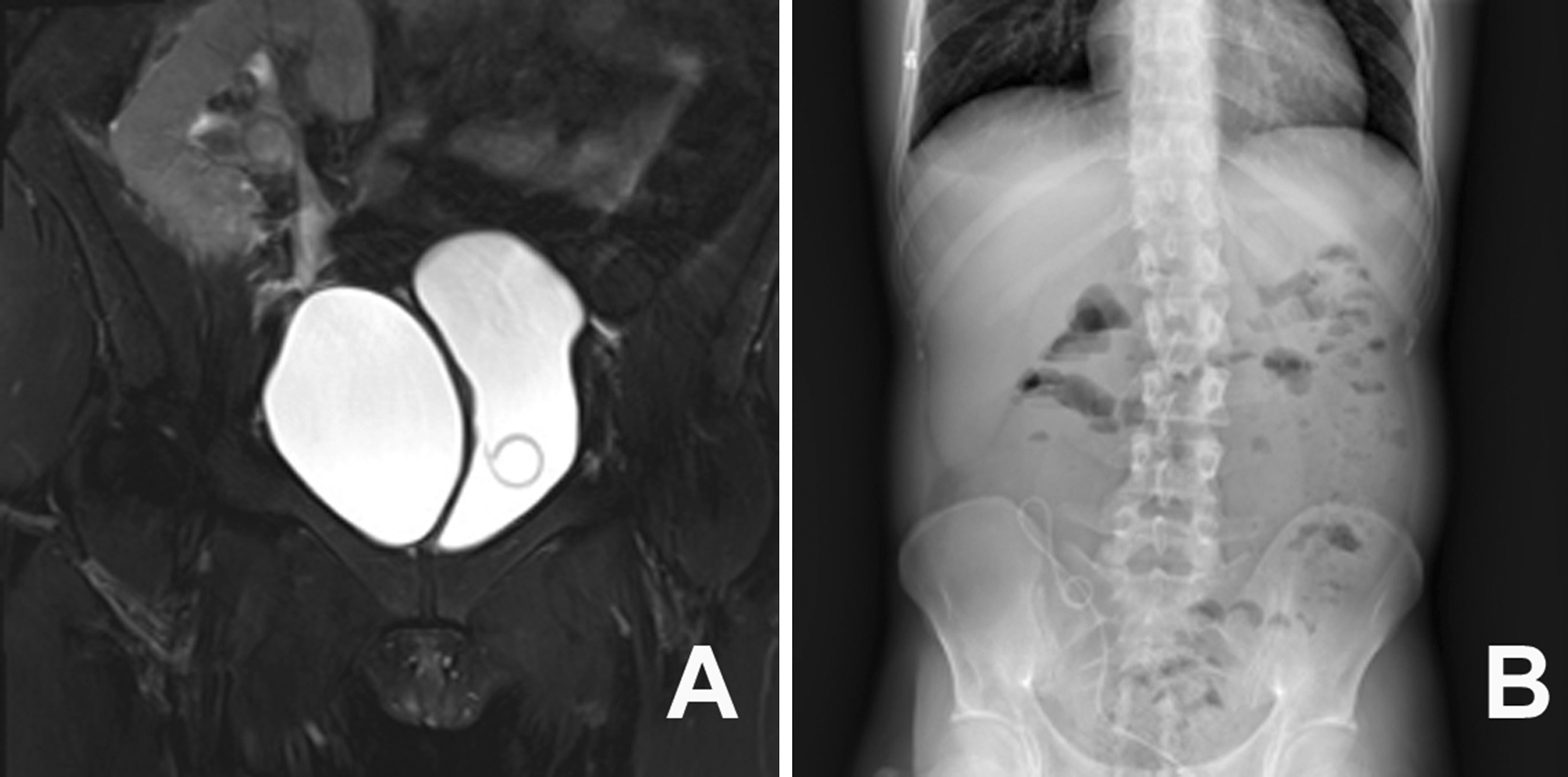
The placement of a double-J stent in a 32-year-old female with a right pelvic lymphocoele compressing the ureter. **a** The placement of a double-J stent into the pelvis of the renal allograft. **b** The KUB film revealed that the stent was inserted into the pelvis

Patient 2 was a 32 years old woman with ESRD secondary to IgA nephropathy, hypertension and renal anemia. This patient was admitted for renal transplantation from a deceased donor on April 2019. The donor was a 38-year-old man who died from traffic accident-related craniocerebral trauma. The donor and recipient were blood-group compatible based on a 5 HLA antigen match, and both flow microcytotoxicity and direct microcytotoxicity cross-match were negative. The panel reactivity for the recipient was 2%. The renal allograft vessels were anastomosed side-to-end to the recipient’s external iliac vessels using two 5/0 prolene running sutures. After placing a double-J stent, the transplanted ureter was anastomosed to the bladder according to the Lich-Gregoire technique using 5/0 polydioxanone sutures. The duration of this procedure was 110 min, with 20 mL of blood loss. The postoperative immunosuppression regimen consisted of tacrolimus, mycophenolate mofetil and prednisolone. At the 14th postoperative day, the double-J stent was removed, and the patient’s s-CR decreased to within the normal level. However, at 20 days after the transplantation, the patient was readmitted due to high fever, oliguria and allograft area pain. The examination of this recipient revealed elevated s-CR, urea nitrogen and uric acid, with electrolyte disturbance. The urine culture grew *Escherichia coli* with positive extended-spectrum beta-lactamase. Ultrasonography revealed a mildly hydronephrotic allograft and complete ureteral dilatation. The patient’s condition improved after intravenous Sulperazone treatment. However, after 1 month, the patient’s s-CR remained elevated during the outpatient test, and the magnetic resonance imaging (MRI) identified a pelvic lymphocoele arising from the right pelvic cavity, which compressed the allograft ureter. Cystoscopic intubation was attempted to decompress the patient’s collecting system prior to performing the PCN, but failed. Based on the successful experience in patient 1, the trans-vesical puncture was repeated to place the double-J stent, and the procedure succeeded. The patient finally recovered her renal function after ultrasound-guided puncture and pelvic lymphocoele drainage. The double-J stent was retained for 3 months, and removed using a cystoscope (Fig. 2).

**Fig. 3:**
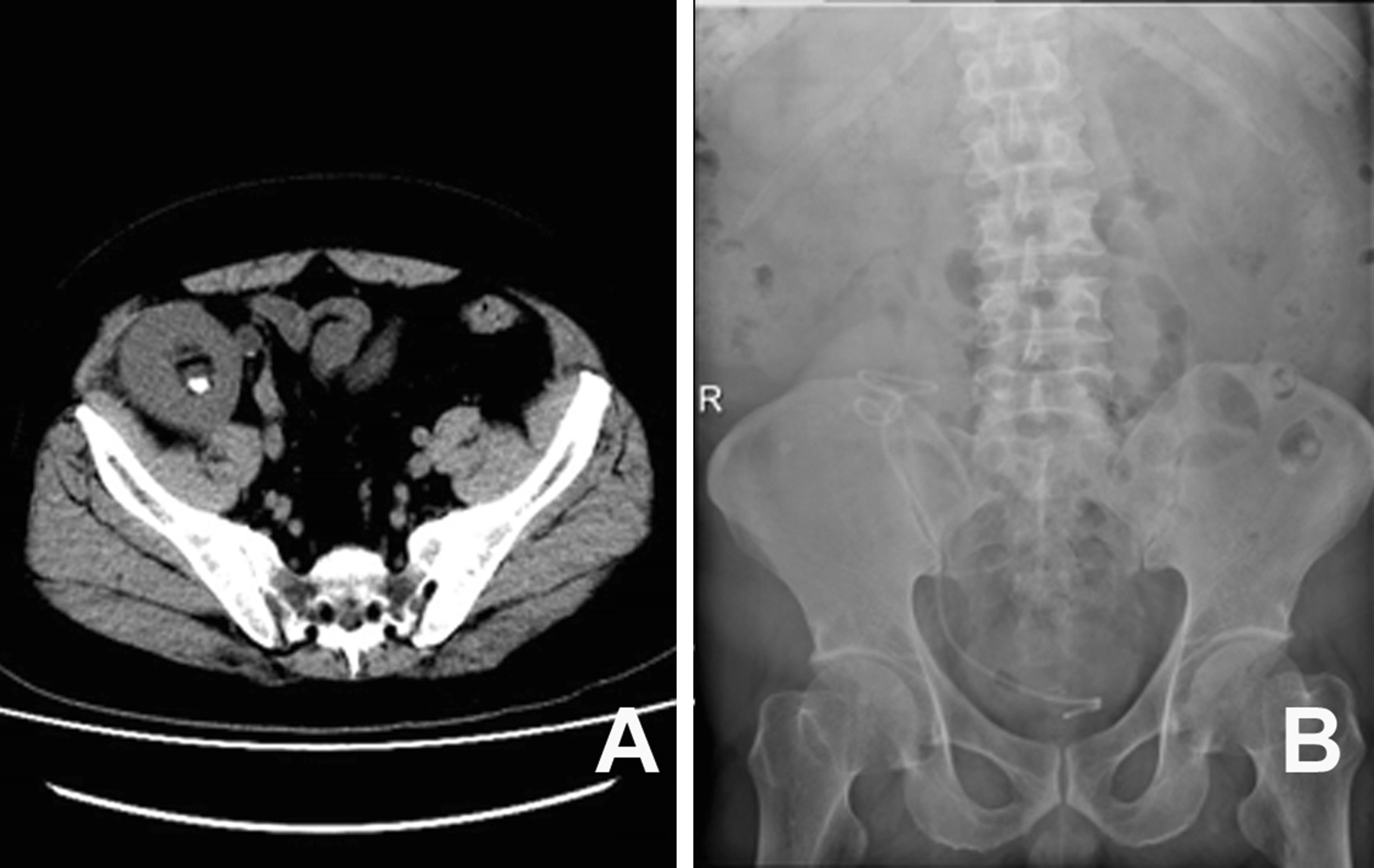
The placement of a double-J stent in a 27-year-old male developing ureterovesical junction stricture and ureteral calculi. **a** The computed tomography revealed multiple transplant ureteral calculi. **b** The KUB film revealed that the double-J stent was inserted into the pelvis

Patient 3 was a 27 years old Chinese male diagnosed with ESRD, who received twice a week of hemodialysis for two years. The patient’s blood type was B (Rh+), which was the same as that of the donor. The donor was the patient’s 52-year-old healthy mother. Ureter and bladder anastomosis were performed using the Lich-Gregoire method. The patient’s postoperative immunosuppression regimen consisted of tacrolimus, mycophenolate mofetil and steroid. At the 14th postoperative day, the double-J stent was smoothly removed using a cystoscope. At discharge, the patient’s renal function returned to normal. At the 22nd postoperative day, the patient was hospitalized due to high fever. The ultrasound merely revealed perirenal effusion, and the patient was discharged after antibiotic treatment. However, at 2 months after the operation, the ultrasound examination revealed a hydronephrotic allograft. Despite the patient’s asymptomatic status, the patient remained hospitalized, and the admission s-CR was slightly elevated at 114 μmol/L, with normal serum urea, uric acid and calcium. The patient’s serum inorganic phosphorus and magnesium levels slightly decreased at 0.67 mmol/L (normal range: 0.85–1.51 mmol/L) and 0.70 mmol/L (normal range: 0.75–1.02 mmol/L), respectively. The patient underwent transurethral cystoscope catheterization under general anesthesia, but failed. The patient was discharged with watchful waiting. At 3.5 months after the transplantation, the patient was admitted for the third time due to fever, oliguria and elevated s-CR. The ultrasonography revealed hydronephrosis with multiple ureteral calculi. The patient underwent cystoscopy with stenting, but failed again due to difficulty of access (the ureteral opening located at the bladder top, near the pubic symphysis) and ureteral opening stenosis. Therefore, trans-vesical puncture was attempted to place the double-J tube again, which was successful. The recovered smoothly after the operation. For the ureteral calculi treatment, ureteroscopy was performed after two weeks. However, the retrograde ureteroscopy through either urethra, or cystostomy access to enter the allograft ureter failed. The investigators resorted to the original approach again for double-J stenting, and was successful. The patient is presently waiting for the lithotripsy (Fig. 3).

## Discussion and conclusions

Renal transplantation is the definitive treatment for patients with ESRD. However, ureteroneocystostomy stenosis and allograft lithiasis are potentially devastating complications [7, 8]. According to recent reports, ureteroneocystostomy stenosis is the most common urologic complication during renal transplantation [8, 9], while allograft lithiasis is another uncommon but difficult-to-treat complication [10]. These complications can lead to renal damage resulting from urinary obstruction and mortality due to infection. Several studies have demonstrated that most cases can be endoscopically managed if patients with hydronephrosis after renal transplantation do not prefer active surveillance or surgery [2, 11, 12]. The timely and effective decompression of obstructed ureters is frequently challenging due to the un-physiologic location of the ureteroneocystostomy [13]. For patients with significant hydronephrosis with renal function decline and sepsis, the prompt insertion of PCN can minimize the morbidity through immediate drainage and decompression, allowing for the diagnosis of the site of obstruction based on antegrade pyelography. However, PCN is associated with a risk of infection, bleeding and graft injuries, causing greater difficulty during the removal and exchange of an indwelling stent. Therefore, it appears more reasonable to use the retrograde ureteral stent placement as a first-line treatment for relieving upper urinary obstruction. Once the retrograde approach fails, the antegrade one can be applied.

**Fig. 4:**
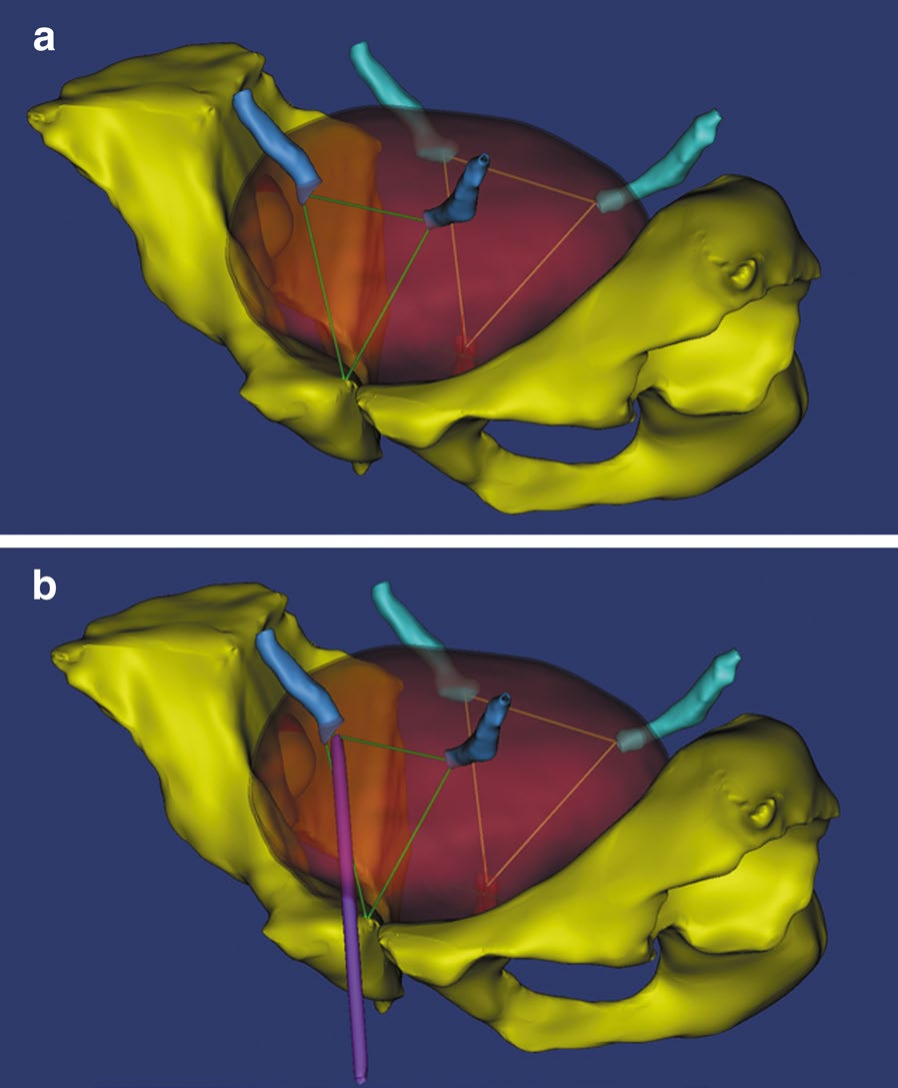
A 3-dimensional graph illustrates the position of the allograft ureteral and original ureteral orifice. **a** The anatomical relationship between the transplant ureteral orifice and the original ureteral orifice upon projection. **b** Introduction of the access by percutaneous suprapubic cystotomy to the ureter orifice of the allograft and the retrograde placement of a double-J stent into the allograft pelvis

**Fig. 5:**
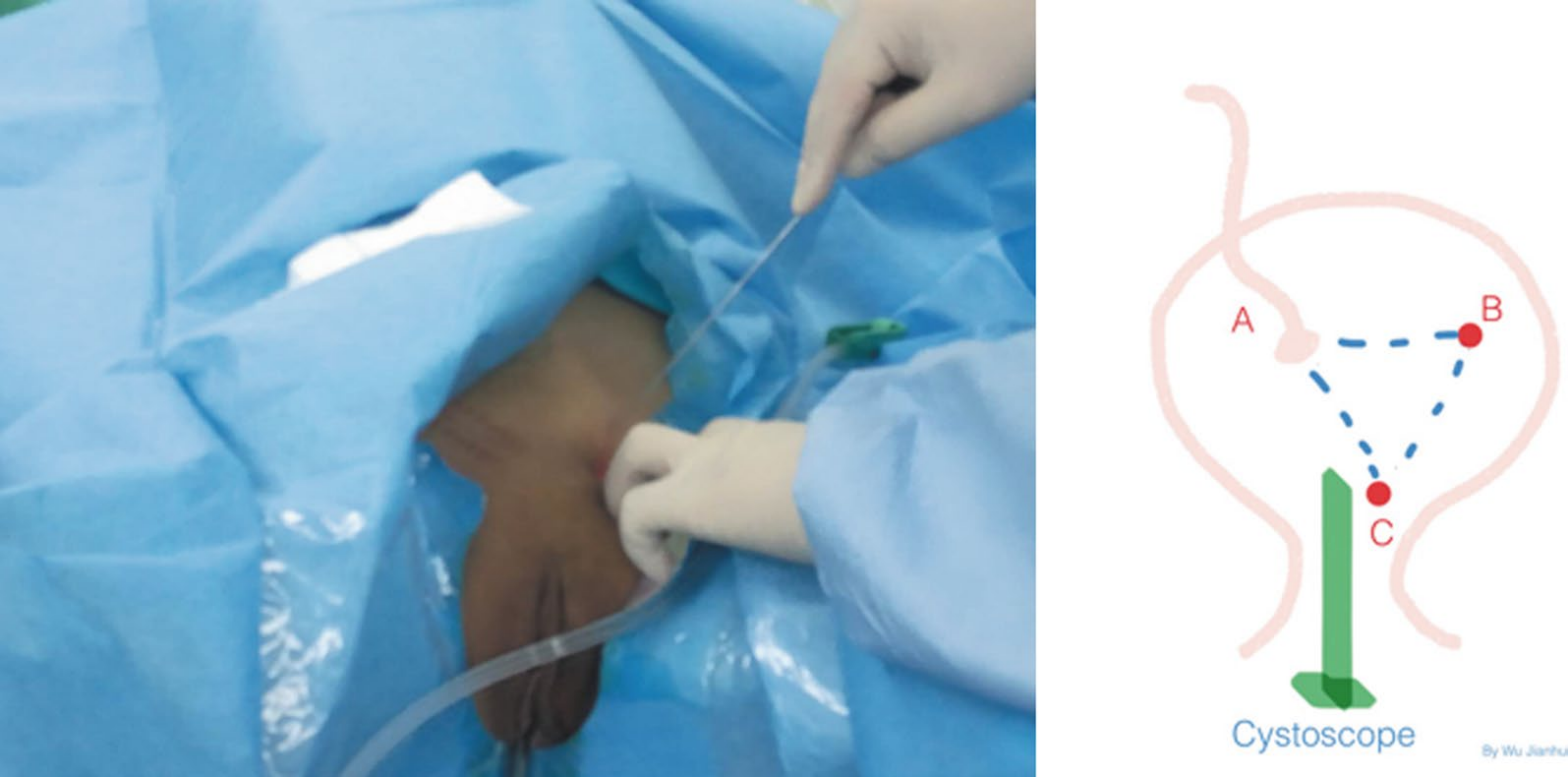
Selection of the puncture site for the suprapubic cystostomy: under cystoscopic monitoring, the location of the vesicostomy puncture site should be selected between points B and C

The retrograde approach proposed by the investigators has its advantages and disadvantages. This approach is minimally invasive, bloodless and repeatable. On the other hand, the success rate of this approach remains unclear, and the main challenge during stent insertion is the localization of the neo-orifice and the difficulty in advancing the stent through the angulated ureteral route [12]. Furthermore, both the bladder outlet and ureteral orifice are usually located in the triangular region plane of the bladder, and this anatomical alignment facilitates the transurethral ureteral intubation due to the same plane and direction. Halstuch et al. [14] reported that during the placement of a double pigtail stent in the native ureter, the stent would constantly be surrounded by the ureteral wall, and any forward pressure applied to the stent promote forward movement. However, the allograft ureter frequently goes into the dome of the bladder at an oblique angle, which makes the ureteral orifice intubation difficult for the following three reasons. First, directing a wire to a ceiling oriented orifice can be challenging. Second, advancing the stent over a guidewire tends to recoil back. Third, the guidewire can easily slide out of the neo-orifice while attempting to pass a ureteral catheter through the wire. At present, few reports have described the retrograde approach of ureteral intubation for transplant kidneys [15]. Elias Hyams et al. reported a retrograde technique for accessing an angle-tipped angiographic catheter. That is, they used a Kumpe or Berenstein catheter to advance a flexible tipped guidewire retrograde into the collecting system under fluoroscopic guidance [16]. From an anatomical perspective, the neo-orifice of the allograft on the right (or left) side of the pelvic cavity resembles the right (or left) ureteral orifice of the native kidney projecting onto the anterior bladder wall (Fig. 4). For instance, in projecting the bladder outlet to the anterior wall of the native bladder, the intubation would be carried out on a triangular-like plane, which is similar to the trigone of the native bladder. In this scenario, the central or left (or right) anterior wall of the native bladder would be selected as the catheter entry site, which is closer and below the neo-orifice (Fig. 5). Using the approach outlined above, a total of five successful RUS-PSABs were performed in three patients with ureteral obstruction after renal transplantation. The investigators consider that two major factors influence the success of the RUS-PSAB, which include a sensor straight tip guidewire (0.035-inch [150 cm]; Boston Scientific, USA) and a double-J tube (Black Silicone Filiform DP Ureteral Stent Set Wire Guide with Hydrophilic Coating, Cook Incorporated, USA).

Zavos et al. [17] reported the ureteropelvic stenosis of renal allografts in 12.5% of transplant recipients, while ureteral obstruction and ureterovesical stricture were found in 28.1% and 30.2% of transplant recipients, respectively. Halstuch et al. [14] reported that the ureterovesical anastomotic stricture had an incidence of 1–4.5% after renal transplantation. Ozkaptan et al. [11] reported that ureterovesical anastomotic obstruction followed by ureteral stenosis was the most common reason for urinary tract stenosis. Etiological factors for ureteral strictures in existing studies include ureteral devascularization, allograft ureterolithiasis, urinary leakage, allograft rejection, ureteral BK virus infection, compression of the ureter by hematoma or lymphoceles, and surgical complications. Ureteral stricture appears to be a common complication in transplant recipients. Indeed, surgeons should provide more attention to the angle between the ureterovesical junction and stricture, and evaluate whether this is too acute. If so, the wire or stent insertion may fail with or without ureteral stenosis. Many ureteroneocystostomy approaches have been described, each with its advantages and disadvantages [8, 18, 19]. The investigators suggest adjusting the vesico-ureteric anastomosis farther away from the suprapubic area after distending the bladder.

There are no practice guidelines for treating post-transplant ureteral stenosis and calculi, and the experience of the urological team has become instrumental. The present case series is the first to describe the approach for the successful RUS-PSAB among patients receiving renal transplantation with ureteral stricture. The experience of the investigators supports the feasibility of the RUS-PSAB approach for allograft obstruction relief. The absence of complications in these three patients was reassuring, but complications from the cystectomy should still be considered. Furthermore, this approach is compatible with procedures, such as ureteroscopic laser lithotripsy. In the third patient, a flexible ureterorenoscopy (F-URS) were planned.

Semi-rigid ureteroscopes were used to attempted to pass through the percutaneous cystotomy access into the transplanted ureter, but eventually failed in the third patient. Sometimes, the operator may succeed in the ureteral intubation through the urethra, similar to that described in the first patient. However, the probability of failure remains high. At present, the investigators suggest the use of the RUS-PSAB method or PCN, since there have been many reports of using flexible ureteroscopes(F-URS) to treat urolithiasis in patients receiving renal transplantation [16, 20–22]. Nonetheless, percutaneous cystotomy access may still offers a clear and accurate access for F-URS, and facilitates laser lithotripsy for managing allograft calculi.

These present results indicate that this novel stent placement technique for allograft recipients is frequently successful. Indeed, this treatment entails higher cost due to the use of disposable materials and percutaneous access. However, the investigators consider the additional cost to be justified by its high success rate, safety and absence of severe consequences, resulting from the failed stent insertion. Therefore, this approach is an attractive alternative for ureteral stent replacement among patients receiving renal transplantation. Finally, the present study is limited by the relatively small cohort size and its single-center nature.

## Supplementary information

**Additional file 1.** Retrograde ureteric stent insertion from percutaneous suprapubic access to the bladder in renal transplant recipients with ureteric stenosis.

## Data Availability

The datasets used and/or analysed during the current study are available from the corresponding author on reasonable request.

## Abbreviations

RT: Renal transplantation
PCN: Percutaneous nephrostomy
MRI: Magnetic resonance imaging
RUS-PSAB: Retrograde ureteral stent insertion through percutaneous suprapubic access to the bladder
F-URS: Flexible ureteroscopes
